# Expectations, concerns, and attitudes regarding whole-genome sequencing studies: a survey of cancer patients, families, and the public in Japan

**DOI:** 10.1038/s10038-022-01100-6

**Published:** 2022-12-12

**Authors:** Izen Ri, Junichi Kawata, Akiko Nagai, Kaori Muto

**Affiliations:** Department of Public Policy, The Institute of Medical Science, The University of Tokyo, Minato-ku, Tokyo Japan

**Keywords:** Ethics, Health policy

## Abstract

Whole-genome sequencing (WGS) is being used in research and clinical settings in cancer genomics. Studies show that cancer patients generally have positive attitudes toward tumor profiling tests; however, few works revealed their attitudes toward WGS. This study clarifies the expectations, concerns, and result preferences of cancer patients (CPs), family members (FMs) and general adults (GAs) regarding WGS study in Japan. We conducted an anonymous survey with 1204 CPs, 5958 FMs, and 2915 GAs in 2021. Despite low awareness of the WGS studies, CPs had the highest expectations for it. FMs had a higher level of concern than CPs and GAs; feeling anxious by knowing the results, being treated unfavorably if germline findings were detected. Both the FMs and CPs were highly concerned about the protection of genetic information. CPs preferred results with actionability, however, only half preferred to know germline findings. Given the possibility of detecting variants across multidisciplinary diseases and the long-term continuity of WGS research, a system is needed in which study participants can consult and receive decision-making support at any time according to their needs.

Whole genome sequencing (WGS), which analyzes entire genomes using next-generation sequencing (NGS), is increasingly introduced in research and clinical settings in cancer genomics.

Several studies have revealed people’s attitudes toward cancer genomics. Advanced cancer patients participate in WGS studies and profiling tests in the hope of gaining new insights into their condition, in addition to improving treatment and contributing to the research [1]. A review indicated that patients generally have positive attitudes toward tumor NGS, and their expectations often exceed the reality of low clinical utility [2]. Patients prefer to know information about cancer and treatable noncancer conditions compared to those with predisposition to untreatable noncancer conditions in whole-exome sequencing (WES), which is one of the comprehensive analysis methods covering protein-coding regions of the genome using NGS [3].

A survey of public attitudes toward WGS study in Japan clarified that those interested in gene-related information had high levels of concern, but this did not affect their willingness to participate in such research, though for people who were not interested in their gene-related information, concerns about WGS negatively impacted their willingness to participate [4]. Another study showed that family members had higher expectations than cancer patients from genomic tumor profiling tests (GTPTs) [5]. However, cancer patients’ attitudes regarding WGS have seldom studied. This study clarifies the expectations, concerns, and result preferences of cancer patients, their families, and the public regarding WGS study, and identifies the issues to be addressed.

Cross-sectional anonymous online surveys were conducted among 5376 cancer patients and family members of cancer patients aged 20–79, and another 35,146 adults in the general Japanese population aged 20–69 in March 2021. These groups were extracted from a database of 3.6 million people compiled by INTAGE Inc. based on national census data or their sub-panel. Cancer patients and family members were registered to the sub-panel as people who were currently going to a hospital for cancer treatment or were living with a person who had undergone cancer treatment within the last year. Before answering, respondents were given a brief explanation of WGS with diagrams, which it analyzed the entire genome, instead of only specific targeted genes as single gene testing (companion diagnostics) and GTPTs.

The number of respondents was 10,731 (response rate: 26.5%). After excluding those aged 70 years or older, the remaining 10,077 respondents were divided into those with a history of cancer (CPs, *n* = 1204), those with family members with cancer (FMs, *n* = 5958), and general adults with no personal history or family history of cancer (GAs, *n* = 2915) based on their responses. It found that 56.6% of CPs, 61.2% of FMs, and 70.6% of GAs had never heard of the WGS study. A total of 30.5% of CPs, 27.2% of FMs, and 18.2% of GAs were willing to participate in the WGS study (Table 1).

**Table 1:** Respondent characteristics, and awareness of and attitudes toward WGS studies

	CPs (*n* = 1204)				FMs (*n* = 5958)				GAs (*n* = 2915)			
	Males		Females		Males		Females		Males		Females	
	*n*	%	*n*	%	*n*	%	*n*	%	*n*	%	*n*	%
Total	504	41.9	700	58.1	2807	47.1	3151	52.9	1719	59.0	1196	41.0
Age group (years)*												
20–29	17	3.4	14	2.0	225	8.0	373	11.8	359	20.9	270	22.6
30–39	21	4.2	47	6.7	374	13.3	573	18.2	366	21.3	253	21.2
40–49	39	7.7	193	27.6	667	23.8	773	24.5	430	25.0	284	23.7
50–59	120	23.8	254	36.3	830	29.6	760	24.1	305	17.7	208	17.4
60–69	307	60.9	192	27.4	711	25.3	672	21.3	259	15.1	181	15.1
Marital status												
Unmarried	87	17.3	133	19.0	936	33.3	905	28.7	776	45.1	396	33.1
Married	417	82.7	567	81.0	1871	66.7	2246	71.3	943	54.9	800	66.9
Do you have any children												
No	171	33.9	270	38.6	1361	48.5	1388	44.0	990	57.6	567	47.4
Yes	333	66.1	430	61.4	1446	51.5	1763	56.0	729	42.4	629	52.6
Education background												
Junior high school	7	1.4	14	2.0	75	2.7	72	2.3	50	2.9	43	3.6
High school	133	26.4	228	32.6	735	26.2	924	29.3	492	28.6	399	33.4
Occupational school	51	10.1	122	17.4	367	13.1	536	17.0	255	14.8	230	19.2
Junior college	7	1.4	147	21.0	48	1.7	608	19.3	34	2.0	186	15.6
University or graduate school	306	60.7	189	27.0	1582	56.4	1011	32.1	888	51.7	338	28.3
Annual household income, JPY												
<3,000,000	110	21.8	168	24.0	541	19.3	837	26.6	427	24.8	360	30.1
3,000,000–5,000,000	124	24.6	195	27.9	706	25.2	904	28.7	432	25.1	339	28.3
5,000,000–7,000,000	99	19.6	141	20.1	511	18.2	611	19.4	352	20.5	228	19.1
7,000,000–10,000,000	96	19.0	128	18.3	628	22.4	524	16.6	336	19.5	197	16.5
≧10,000,000	75	14.9	68	9.7	421	15.0	275	8.7	172	10.0	72	6.0
Awareness of genetic testing												
Understand what it means	138	27.4	170	24.3	658	23.4	684	21.7	237	13.8	181	15.1
Have heard of it	318	63.1	480	68.6	1809	64.4	2152	68.3	1055	61.4	770	64.4
Have never heard of it	48	9.5	50	7.1	340	12.1	315	10.0	427	24.8	245	20.5
Experience with genetic testing												
Have taken it	51	11.2	63	9.7	76	3.1	81	2.9	33	2.6	27	2.8
Have never taken it	395	86.6	580	89.2	2363	95.8	2716	95.8	1225	94.8	904	95.1
Don’t recall	10	2.2	7	1.1	28	1.1	39	1.4	34	2.6	20	2.1
Awareness of WGS studies												
Know about it	89	17.7	46	6.6	380	13.5	188	6.0	148	8.6	44	3.7
Have heard of it	173	34.3	215	30.7	941	33.5	797	25.3	445	25.9	221	18.5
Have never heard of it	242	48.0	439	62.7	1486	52.9	2166	68.7	1126	65.5	931	77.8
Participation in WGS studies												
Want to participate	163	32.3	204	29.1	856	30.5	765	24.3	331	19.3	199	16.6
Don’t want to participate	107	21.2	138	19.7	614	21.9	693	22.0	579	33.7	384	32.1
Cannot decide	234	46.4	358	51.1	1337	47.6	1693	53.7	809	47.1	613	51.3

*WGS* Whole genome sequencing, *CPs* cancer patients, *FMs* family members of cancer patients, *GAs* general adults

*The mean age (males/females) was 58.6/52.9 years for CPs, 49.7/47.2 years for FMs, 43.1/42.5 years for GAs, and 48.4/46.9 years overall. The median age (males/females) was 62.0/53.0 years for CPs, 51.0/48.0 years for FMs, 43.0/42.0 years for GAs, and 50.0/48.0 years overall

Expectations of CPs were the highest among the three groups for all items, especially for diagnosis, treatment, managing the health of their family, and advances in medicine through development of database (Fig. 1a). GAs expectations were lower than those of CPs and FMs. Among CPs, 74.7% (the sum of “somewhat agree” and “agree”) expected it to be beneficial in diagnosing their disease, 79% expected it to lead to a cure, and 75.7% expected it to lead to the development of medicine by building a large-scale database.

**Fig. 1:**
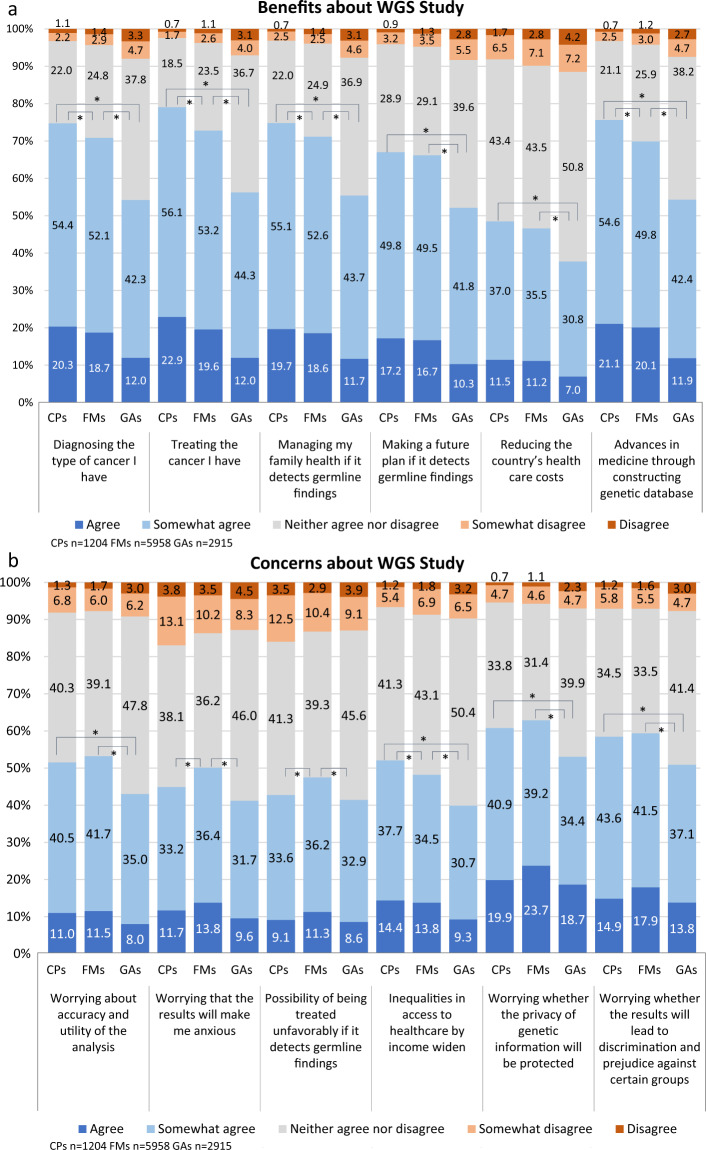
Perception of benefits and concerns about WGS study. * Indicates statistical significance for the sum of “agree” and “somewhat agree” (*p*  <  0.05). The Bonferroni method is used in the adjustment of multiple comparisons. 5-point Likert scale was used to measure the respondents’ perception of benefits (**a**) and concerns (**b**) about WGS study. WGS Whole genome sequencing, CPs cancer patients, FMs family members of cancer patients, GAs general adults

Approximately 60% of both CPs and FMs were concerned about the privacy of genetic information will be protected and the possibility of discrimination and prejudice against certain groups, while about 50% of them were concerned about the accuracy and utility of WGS (Fig. 1b). FMs were especially more worried than CPs and GAs about the results making them anxious, and the possibility of being treated unfavorably if germline findings were detected.

Regarding result disclosure preference of those who answered that they would like to participate in the WGS study (Fig. 2), CPs were most interested in information that would lead to the diagnosis and treatment of their disease (85.3%), and FMs were in the possibility of developing preventable or treatable disease (80.9%). About 70% of both CPs and FMs wanted to know about life-threatening and urgent diseases. The possibility of developing non-preventable or untreatable diseases was less preferred to that of preventable or treatable diseases. A total of 54.5% of CPs and 59.8% of FMs wanted to know germline findings.

**Fig. 2:**
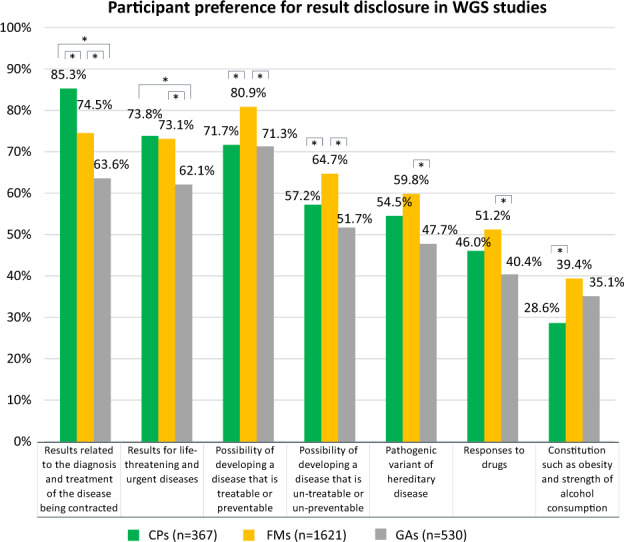
Participant preference for result disclosure in WGS studies * Indicates statistical significance (*p* < 0.05). The Bonferroni method is used in the adjustment of multiple comparisons. Multiple answer selection was used to measure the respondents’ preference for result disclosure in WGS studies. WGS Whole genome sequencing, CPs cancer patients, FMs family members of cancer patients, GAs general adults

Although CPs preferred to know the results with clinical utility or actionability, only about half of them preferred to know inherited diseases. This is in contradiction with a previous study that about 86% of CPs were interested in germline findings in the tumor profiling test [6], and about 70% of CPs opted for such results in WES in Japan [7]. These findings may contribute to the ongoing discussion about whether secondary findings (SFs) should be revealed to patients. Since patients’ preferences on this were influenced by their disease experience, knowledge, and life context [8, 9], the process of such disclosure is suggested to be tailored to a patient’s individual circumstances [10]. Careful consideration should be given to the benefits and burdens of knowing SFs on conditions that are not related to the patient’s current illness or symptoms, so they can make individual choices.

This study has several limitations. It could not provide a specific description of WGS study and a price for clinical test when the study translated to clinical practice. It should be noted that the survey was conducted with low awareness of WGS study. In addition, qualitative research and dialogue are needed to explore how people distinguish WGS from existing genetic testing, and the specific reasons regarding their expectations and concerns. Despite such limitations, this survey is the first to show the attitudes of CPs and FMs in Japan.

CPs had higher expectations for WGS study leading to diagnosis and treatment, as compared to the previous survey on GTPT which was about 50% [5]. This is presumably a reflection of the high expectations from novel technologies. Thus, explaining the limitations in clinical utility, and the probability of reaching a diagnosis or treatment to avoid excessive expectations or “diagnostic and therapeutic misconception” is required. In addition, the scheme of informed consent and genetic counseling based on two points in time of pre/post testing may not be suitable for a nationwide, long-term WGS study. For example, in a survey of participants in the UK’s 100,000 Genomes Project, approximately 20% of cancer patients did not recall their initial consent decision for disclosure of additional findings [11]. A survey of GTPT institutions in Japan showed that although almost all of participants preferred disclosing SFs, when presumed germline pathogenic variants (PGPVs) were detected, only about 20% of them proceeded to confirmatory testing because they prioritized their cancer treatment [12]. In WGS study, variants may be detected across multidisciplinary disease, and the interpretations may change through the study development. Given that study participants may not remember what they gave consent to, and their life circumstances and life stages may change over the years, portal sites that enable them to check study progress and key details, and consulting services that allow them to seek support from experts in decision-making at any time are required.

As both CPs and FMs were highly concerned about the genetic privacy, ensuring transparency and infrastructure for secure access to data are crucial. Creating an infrastructure that promotes the use of data provided by the participants while ensuring their rights of access and proactive control over them, as proposed by the European Health Data Space (EHDS) released by the European Commission [13] is one way to ensure it.

FMs had concerns about being treated unfavorably based on genetic characteristics, while there are no laws or regulations against genetic discrimination in Japan [14, 15]. Several studies showed despite the implementation of The Genetic Information Nondiscrimination Act (GINA) in the US, low awareness and insufficient understanding persists [16, 17]; even in a survey conducted 10 years later, the respondents answered incorrectly and indicated that they would refuse genetic testing due to fairness of discrimination in employment and insurance [18]. It suggests that mere enactment of legislation will not necessarily alleviate or dispel their concerns. Steps should be taken to inform the public and organizations involved to prevent disadvantages. An international systematic review revealed that several contexts exist for genetic discrimination; insurance, employment, familial, social, and public sector [19]. Adverse treatment such as employment and insurance, can be prevented to some extent by law or regulation, while social relations, stigmatization, and prejudice, are more subtle and difficult to prove or prevent. Ongoing fears of discrimination are the possibility of re-identifying genetic relatives of the data donor from the database, and the presence of genetic markers of disease within specific population groups, which will be used to stigmatize an already vulnerable population [20]. Although it is beyond the scope of this study, further investigation is needed to explore in detail the perceptions and experiences of those who participate in WGS study.

## Supplementary information

Supplemental Figure
