# Correction: Expectations, concerns, and attitudes regarding whole-genome sequencing studies: a survey of cancer patients, families, and the public in Japan

**DOI:** 10.1038/s10038-022-01108-y

**Published:** 2022-12-20

**Authors:** Izen Ri, Junichi Kawata, Akiko Nagai, Kaori Muto

**Affiliations:** Department of Public Policy, The Institute of Medical Science, The University of Tokyo, Minato-ku, Tokyo Japan

**Keywords:** Ethics, Health policy

Correction to: *Journal of Human Genetics* 10.1038/s10038-022-01100-6, published online 12 December 2022

In the abstract part, “5968 FMs” should be “5958 FMs”, and “those with family members with cancer (FMs, *n* = 5968)” in the fifth paragraph should be “(FMs, *n* = 5958)”.

The original article has been corrected.

