# Supplementary material for: Expectations, concerns, and attitudes regarding whole-genome sequencing studies: a survey of cancer patients, families, and the public in Japan
Source: J Hum Genet. 2022 Dec 12;68(4):281–5. doi: 10.1038/s10038-022-01100-6 (PMC10040335; doi:10.1038/s10038-022-01100-6)
Supplement: Supplementary file 1 — Supplemental Figure [file 10038_2022_1100_MOESM1_ESM.pdf]

Supplemental Figure. Distribution of perception of benefits and concerns about WGS study stratified by sex and age

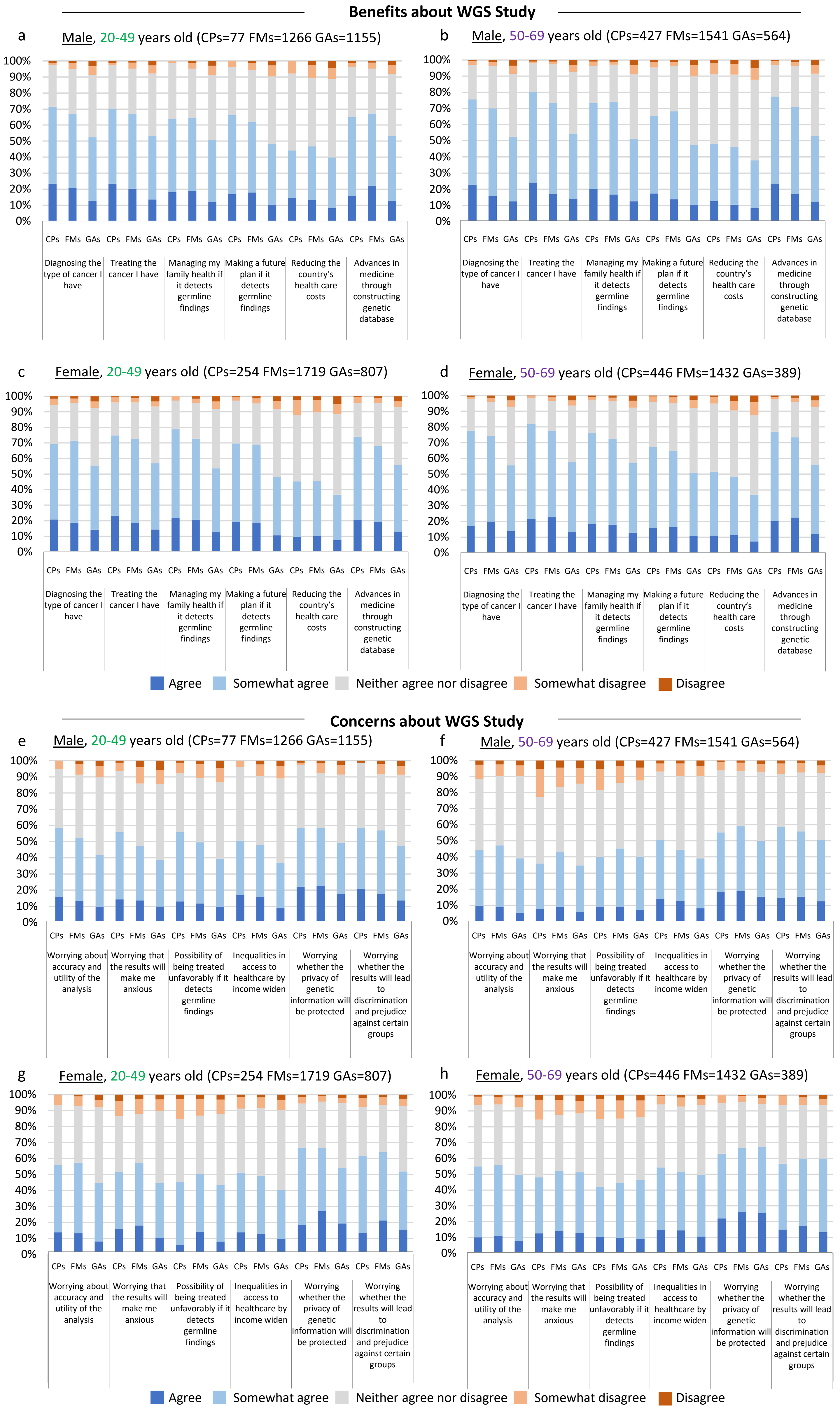

Distribution of perception of benefits and concerns about WGS stratified by sex and age. All groups stratified sex and age (50< or 50≥). 5-point Likert scale was used to measure the respondents' perception of benefits (a-d) and concerns (e-h) about WGS study. *WGS* Whole genome sequencing, *CPs* cancer patients, *FMs* family members of cancer patients, *GAs* general adults.
